# Remnant cholesterol and cardiovascular risk in maintenance haemodialysis

**DOI:** 10.1093/ckj/sfag337

**Published:** 2026-09-25

**Authors:** Diego Moriconi, Marco Ulivelli, Adamasco Cupisti, Domenico Giannese, Vincenzo Cantaluppi, Massimiliano Migliori, Vincenzo Panichi, the RISCAVID group, Giovanni Manca Rizza, Sabrina Paoletti, Emanuela Mangione, Aldo Casani, Laura Bozzoli, Giovanni Grazi, Valentina Batini

**Affiliations:** Department of Clinical and Experimental Medicine, University of Pisa, Italy; Department of Clinical and Experimental Medicine, University of Pisa, Italy; Department of Clinical and Experimental Medicine, University of Pisa, Italy; Nephrology and Dialysis Unit, AOUP, Pisa, Italy; Nephrology and Kidney Transplantation Unit, Department of Translational Medicine, University of Piemonte Orientale (UPO), “Maggiore Della Carità” University Hospital, Novara, Italy; Nephrology and Dialysis Unit, Versilia Hospital, Italy; Department of Clinical and Experimental Medicine, University of Pisa, Italy

**Keywords:** cardiovascular risk, geriatric nutritional risk index, haemodialysis, mortality, remnant cholesterol

## Abstract

**Background:**

Remnant cholesterol (remnant-C) has emerged as a marker of residual cardiovascular risk beyond low-density lipoprotein cholesterol (LDL-C) but data in haemodialysis populations remain limited. We investigated the association of remnant-C with cardiovascular and mortality outcomes in maintenance haemodialysis patients.

**Methods:**

This post-hoc analysis included 624 patients from the prospective RISCAVID cohort. Remnant-C was calculated as total cholesterol minus high-density lipoprotein cholesterol minus directly measured LDL-C. The primary endpoint was 3-point major adverse cardiovascular events (MACE), defined as cardiovascular death, nonfatal myocardial infarction, or nonfatal ischaemic stroke. All-cause mortality was analysed as a secondary endpoint. Associations were assessed using multivariable Cox regression models.

**Results:**

During a median follow-up of 31 months, 328 patients experienced a 3-point MACE and 375 died. Remnant-C was independently associated with 3-point MACE after adjustment for age, sex, traditional cardiovascular risk factors, LDL-C, and statin therapy (HR 1.06 per 10 mg/dl increase, 95% CI 1.01–1.11). The association remained significant after additional adjustment for the Geriatric Nutritional Risk Index (GNRI) and normalized protein catabolic rate. Restricted cubic spline analysis showed a progressive increase in cardiovascular risk across increasing remnant-C concentrations without evidence of a nonlinear threshold effect. For all-cause mortality, neither remnant-C nor LDL-C retained prognostic significance after multivariable adjustment. Conversely, GNRI was independently associated with mortality risk, whereas it was not associated with cardiovascular events.

**Conclusion:**

In maintenance haemodialysis patients, remnant-C was independently associated with major cardiovascular events but not with all-cause mortality, whereas the GNRI was associated with mortality but not cardiovascular outcomes. These findings suggest distinct contributions of residual atherogenic risk and nutritional status to cardiovascular and survival outcomes in end-stage kidney disease.

## Graphical Abstract

**Graphical Abstract:**
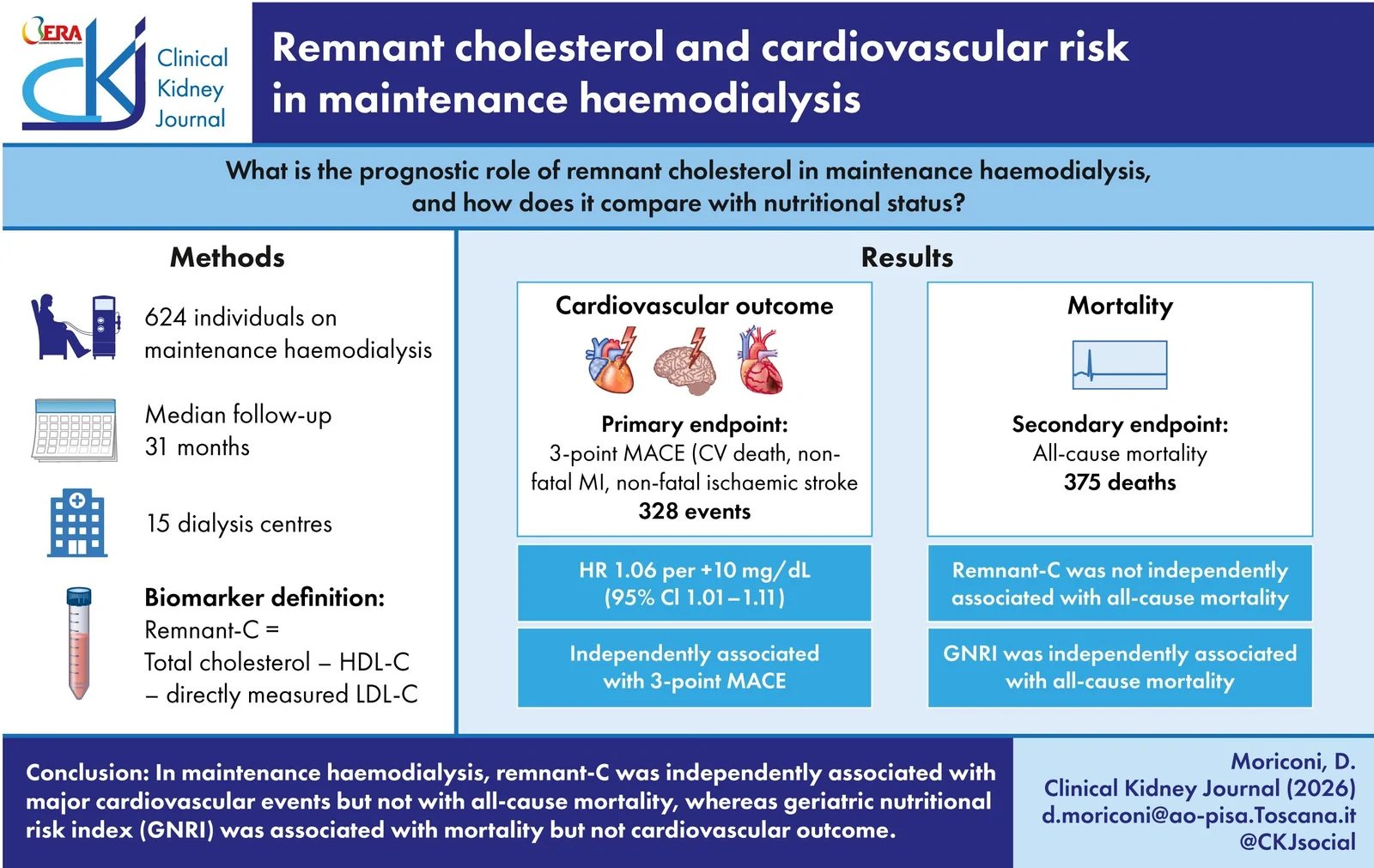

KEY LEARNING POINTS **What was known:**

- Patients receiving maintenance haemodialysis have an extremely high cardiovascular risk.
- LDL cholesterol has limited prognostic value in dialysis populations.
- Remnant cholesterol has emerged as a marker of residual cardiovascular risk, but evidence in haemodialysis remains scarce.

**This study adds:**

- Remnant cholesterol was independently associated with major adverse cardiovascular events.
- The association persisted after adjustment for LDL cholesterol, statin therapy, and nutritional status.
- Remnant cholesterol was not independently associated with all-cause mortality.

**Potential impact:**

- Remnant cholesterol may help identify residual cardiovascular risk beyond LDL cholesterol in haemodialysis patients.
- Cardiovascular events and mortality appear to reflect distinct biological pathways.
- Combined assessment of atherogenic burden and nutritional status may improve risk stratification.

## INTRODUCTION

Patients with end-stage kidney disease (ESKD) undergoing dialysis have markedly elevated cardiovascular risk. Cardiovascular disease (CVD) remains the leading cause of morbidity and mortality, accounting for nearly half of all deaths despite advances in preventive and therapeutic strategies [1]. This excess risk reflects the interplay of accelerated atherosclerosis, chronic inflammation, vascular calcification, and protein-energy wasting, which contribute to the unique cardiovascular phenotype of advanced kidney disease [2, 3].

Although elevated low-density lipoprotein cholesterol (LDL-C) is a major cardiovascular risk factor and primary target of lipid-lowering therapy in the general population, its prognostic value appears attenuated in patients undergoing dialysis. Randomized trials of statin therapy in haemodialysis populations have shown limited cardiovascular benefit compared with that observed in non-CKD populations, suggesting that LDL-centred paradigms may incompletely capture cardiovascular risk in ESKD [4–6].

Moreover, dialysis patients exhibit a characteristic dyslipidaemic profile marked by elevated triglycerides (TG), reduced high-density lipoprotein cholesterol (HDL-C), and relatively normal LDL-C levels. These abnormalities are largely driven by impaired clearance of triglyceride-rich lipoproteins, promoting the accumulation of highly atherogenic remnant particles [4, 7].

In recent years, increasing attention has focused on remnant cholesterol (remnant-C), defined as the cholesterol content of triglyceride-rich lipoproteins and their remnants [8]. Experimental, epidemiological and genetic studies suggest that remnant-C contributes to residual atherothrombotic risk independently of LDL-C, particularly in metabolic conditions characterized by hypertriglyceridaemia, diabetes and persistent cardiovascular risk despite controlled LDL-C levels [9, 10]. Unlike LDL particles, remnant lipoproteins are cholesterol-rich and can directly penetrate the arterial wall promoting foam-cell formation, vascular inflammation, and plaque instability [11, 12]. Observational studies have shown that elevated remnant-C is associated with major adverse cardiovascular events (MACE) independently of LDL-C and other lipid parameters, whereas LDL-C does not consistently retain prognostic significance [10, 13].

Recent evidence has extended these observations to individuals with impaired kidney function. In 10 427 participants with an eGFR <60 ml/min/1.73 m² from the Copenhagen General Population Study, higher remnant-C was associated with an increased risk of atherosclerotic cardiovascular disease (ASCVD), whereas LDL-C did not retain a comparable prognostic association. However, patients receiving maintenance dialysis were excluded. Therefore, whether remnant-C retains prognostic value in the unique metabolic and nutritional milieu of haemodialysis remained uncertain [14].

A recent nationwide Korean cohort including more than 20 000 dialysis patients demonstrated an independent association between elevated remnant-C and myocardial infarction, while no significant relationship was observed with ischaemic stroke [15]. These findings support the hypothesis that remnant-C may represent a contributor to cardiovascular risk in ESKD beyond traditional lipid markers.

Nevertheless, important questions remain regarding the interaction between lipid-related risk and nutritional status. Interpretation of lipid biomarkers in dialysis patients is complicated by reverse epidemiology, whereby lower cholesterol levels may paradoxically reflect malnutrition, inflammation, frailty or protein-energy wasting and associate with worse outcomes. The Geriatric Nutritional Risk Index (GNRI), a validated nutritional marker in dialysis populations, provides prognostic information beyond traditional cardiovascular risk factors and may influence all-cause mortality [16, 17].

On this basis, the present study aimed to evaluate the association between remnant-C and cardiovascular outcomes in a cohort of patients receiving maintenance dialysis. In addition, we sought to investigate whether remnant-C retained prognostic significance independently of LDL-C and whether nutritional status differentially influenced cardiovascular and mortality risk within the ESKD population.

## MATERIALS AND METHODS

### Study cohort

This work is a post-hoc analysis of the previously collected data from the prospective and observational study registered in the Cochrane Renal Group registry as ‘Cardiovascular risk in dialysis: RISCAVID study’ (#CRG040700112) [18].

A total of 753 patients with ESKD receiving maintenance haemodialysis for at least three were in the RISCAVID cohort and followed up to 84 months, from 15 dialysis facilities in north-western Tuscany (Italy). Of the 753 patients originally enrolled in the RISCAVID cohort, 129 were excluded because one or more lipid measurements required for remnant-C calculation, particularly directly measured LDL-C, were unavailable. The final analysis therefore included 624 patients with calculable remnant-C. No data imputation was performed.

Clinical and dialysis-related data were systematically collected, including dialysis vintage, vascular access type, dialysis modality, and adequacy parameters. Information regarding the underlying cause of ESKD was also recorded for all participants.

### Cardiovascular outcomes

Fatal and nonfatal events were assessed at 6-month intervals throughout the 84-month follow-up period. Both fatal and nonfatal events were recorded and adjudicated as cardiovascular (myocardial infarction, congestive heart failure, stroke, sudden cardiac death) or noncardiovascular (infection, malignancy, other, or unknown causes) ones.

The primary cardiovascular endpoint of the present analysis was a composite 3-point major adverse cardiovascular event (3-point MACE), defined as the composite of cardiovascular death, nonfatal myocardial infarction and nonfatal ischaemic stroke. Myocardial infarction was defined according to clinical presentation, serial electrocardiographic changes and cardiac enzyme elevation. Ischaemic stroke was defined as the occurrence of new focal neurological deficits with radiological confirmation. Cardiovascular events were independently adjudicated by two physicians not otherwise involved in the study.

### Laboratory and nutritional assessments

Blood samples were obtained on a midweek dialysis day at fasting and after 20–30 min rest in the semirecumbent position.

Routine biochemistry was assayed locally using standard laboratory methods.

LDL-C was directly measured using an enzymatic in order to minimize potential inaccuracies related to hypertriglyceridaemia and altered lipoprotein metabolism in ESKD patients. Remnant cholesterol was calculated as total cholesterol minus HDL cholesterol minus LDL cholesterol.

Serum albumin was measured by nephelometry (Dade Behring GmbH, Marburg, Germany; intra- and inter-assay CV 4.3% and 4.4%, respectively) and high-sensitivity CRP by a modified high-sensitivity laser nephelometry assay (Behring Diagnostics GmbH, Marburg, Germany), as previously described [18].

Kt/V was calculated using the second-generation single-pool Daugirdas formula [19].

Normalized protein catabolic rate (nPCR) was calculated using the midweek formula by Depner and Daugirdas [18].

Body weight at the end of the dialysis session was assumed to calculate normalized nPCR and body mass index (BMI).

Nutritional status was assessed using the GNRI, calculated as 14.89 × serum albumin (g/dl) + 41.7 × (body weight/ideal body weight), with ideal body weight defined as 22 × height² (m²) and the weight ratio capped at 1 when body weight > ideal body weight [17].

### Statistical analysis

Continuous variables are reported as mean ± standard deviation or median [interquartile range], as appropriate according to data distribution, whereas categorical variables are presented as counts and percentages. Comparisons across remnant-C tertiles were performed using one-way analysis of variance (ANOVA), Kruskal–Wallis test or χ² test, as appropriate.

Survival analyses were performed using Cox proportional hazards regression models. Remnant-C was analysed both as a continuous variable (per 10 mg/dl increase) and according to tertiles derived from its distribution within the study population. Variables considered clinically relevant or significantly associated with outcomes at univariate analyses were included in multivariable models. Hazard ratios (HRs) and 95% confidence intervals (CIs) were reported.

Adjusted survival curves were estimated from multivariable Cox regression models.

Potential nonlinearity in the association between remnant-C and 3-point MACE was explored using a natural cubic spline transformation of remnant-C with 4 degrees of freedom within a Cox proportional hazards model. The model was adjusted for age, sex, BMI, diabetes, hypertension, smoking status, previous cardiovascular events, LDL-C, and statin therapy. Adjusted hazard ratios with 95% CIs were plotted across the observed remnant-C range, using 30 mg/dl as the reference value.

A two-sided *P*-value <.05 was considered as statistically significant.

Statistical analyses were performed using JMP Pro version 18 (SAS Institute, Cary, NC, USA).

## RESULTS

### Baseline characteristics according to remnant cholesterol tertiles

A total of 624 patients on haemodialysis were included in the study population and stratified according to remnant-C tertiles (lower, middle and higher remnant-C tertiles; *n* = 208 for each group). The remnant-C concentrations resulted 18 ± 4 mg/dl in the lower tertile, 31 ± 4 mg/dl in the middle tertile and 57 ± 22 mg/dl in the higher tertile.

Remnant-C serum levels showed a right-skewed distribution within the study population, with progressive separation across tertile cut-offs (Supplementary Fig. S1A).

**Figure 1:**
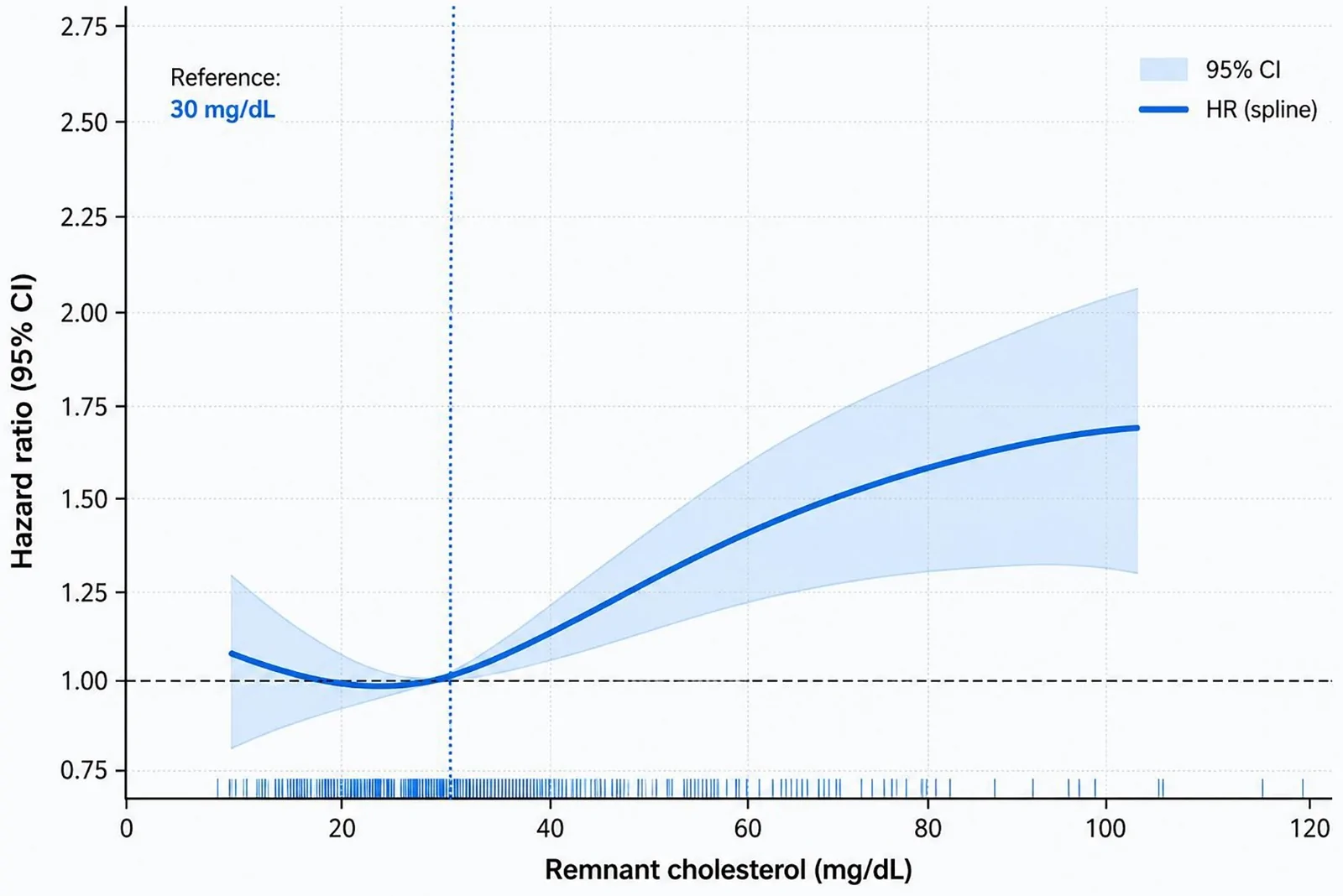
Association between remnant cholesterol and risk of 3-point major adverse cardiovascular events (MACE). Restricted cubic spline analysis showing the adjusted hazard ratio (HR) for 3-point MACE according to remnant cholesterol concentrations. The model was adjusted for age, sex, BMI, diabetes mellitus, hypertension, smoking status, previous cardiovascular disease, LDL-C, and statin therapy. Hazard ratios are shown relative to a reference remnant cholesterol value of 30 mg/dl. Shaded areas represent 95% confidence intervals. Distribution of remnant cholesterol values within the study population is shown by rug plots along the *x*-axis..

Baseline characteristics across remnant-C tertiles are summarized in Table 1. Patients of the lower remnant-C tertile were slightly older compared with those of the middle and higher tertiles, whereas gender distribution was similar across groups, with women accounting for ∼38% of the overall population.

**Table 1:** Baseline characteristics of the study population according to remnant cholesterol (remnant-c) tertiles.

	Lower remnant-C *N* = 208	Middle remnant-C *N* = 208	Higher remnant-C *N* = 208	*P*-value
Age, years	68 ± 13^a^	66 ± 14	65 ± 13	.045
Sex, F (%)	71 (34)	85 (41)	82 (39)	.328
Weight, kg	63 ± 11^a,b^	66 ± 13*	70 ± 13	<.001
BMI, kg/m^2^	22.7 ± 3.7^a,b^	23.7 ± 4.0*	25.1 ± 4.1	<.001
Smoking				.072
Current, *n* (%)	56 (27)	44 (23)	60 (29)	
Ex, *n* (%)	38 (18)	22 (11)	26 (12)	
No, *n* (%)	114 (55)	138 (66)	122 (59)	
Kidney disease				.423
*Congenital/Hereditary*	22 (10.6)	31 (14.9)	26 (12.5)	
*Primary*	40 (19.2)	45 (21.6)	44 (21.2)	
*Secondary*	20 (9.6)	18 (8.7)	19 (9.1)	
*Diabetic*	16 (7.7)	25 (12.0)	30 (14.4)	
*Vascular*	40 (19.2)	28 (13.5)	33 (15.9)	
*Uncertain aetiology*	70 (33.7)	61 (29.3)	56 (26.9)	
Hypertension, *n* (%)	122 (58.7)	105 (50.5)	125 (60.1)	.103
SBP, mmHg	137 ± 21	134 ± 20	135 ± 22	.548
DBP, mmHg	74 ± 10	74 ± 9	75 ± 11	.713
Type-2-Diabetes, *n* (%)	29 (13.4)^a,b^	35 (16.8)^a^	55 (26.4)	.007
History CVD, *n* (%)	29 (13.9)	30 (14.4)	31 (14.9)	.962
Previous stroke, *n* (%)	6 (2.9)	12 (5.8)	16 (6.7)	.098
Statin therapy, *n* (%)	75 (36.1)^a,b^	46 (22.1)^a^	35 (16.8)	<.001
Total cholesterol, mg/dl	145 ± 38^a,b^	164 ± 42^a^	194 ± 48	<.001
HDL cholesterol, mg/dl	50 ± 16^a,b^	41 ± 12	40 ± 11	<.001
LDL cholesterol, mg/dl	78 ± 34^a,b^	93 ± 48	97 ± 40	<.001
Triglycerides, mg/dl	91 [75–106]^a,b^	151 [138–169]^a^	252 [215–318]	<.001
Remnant-C, mg/dl	18 ± 4^a,b^	31 ± 4^a^	57 ± 20	<.001
Dialysis technique				.614
*HDF*	97 (46.6)	107 (51.4)	101 (48.6)	
*HD*	111 (53.4)	101 (48.6)	107 (51.4)	
Kt/V	1.39 ± 0.26	1.38 ± 0.24	1.40 ± 0.25	.727
Dialysis vintage, months	42 [18–89]	48 [24–96]	40 [19–78]	.177
Vascular access				.102
*AVF, n (%)*	185 (88.9)	189 (91.3)	197 (94.7)	
*CVC, n (%)*	23 (11.1)	18 (8.7)	11 (5.3)	
nPCR, g/kg/die	1.06 ± 0.25	1.12 ± 0.27	1.10 ± 0.27	.099
Haemoglobin, g/dl	11.5 ± 1.45^a,b^	11.9 ± 1.50	11.9 ± 1.40	.005
Serum albumin, g/dl	3.58 ± 0.47^a,b^	3.71 ± 0.42	3.74 ± 0.43	.002
GNRI	90 ± 12^a,b^	93 ± 12	95 ± 10	<.001
hs-CRP, mg/l	4.2 [1.7–8.6]	4.9 [2.1–9.3]	4.3 [2.3–7.9]	.777

^a^  *vs* High remnant-C.

^b^  *vs* Medium remnant-C.

Patients in the higher remnant-C exhibited a progressively less favourable metabolic profile, particularly when compared with the lower remnant-C tertile. Specifically, higher remnant-C group showed significantly higher total cholesterol (194 ± 48 vs. 145 ± 38 mg/dl), LDL-C (97 ± 40 vs. 78 ± 34 mg/dl), and triglyceride (252 [215–318] vs. 91 [75–106] mg/dl) serum levels, together with markedly lower HDL-C levels (40 ± 11 vs. 50 ± 16 mg/dl) compared with the lower remnant-C tertile (all *P* < .001) (Table 1).

Despite this metabolic clustering, remnant-C and LDL-C showed only a modest correlation within the study population (Supplementary Fig. S1B).

The prevalence of type 2 diabetes mellitus progressively increased across remnant-C tertiles (13.4%, 16.8%, and 26.4% in the lower, middle and higher tertiles, respectively; *P* = .007). In contrast, statin therapy showed an inverse graded distribution, progressively decreasing from the lower to the higher remnant-C tertile (36.1%, 22.1%, and 16.8%, respectively; *P* < .001). No significant differences were observed with regard to smoking status, hypertension prevalence, previous CVD, dialysis modality, and adequacy parameters (Table 1).

Nutritional and laboratory parameters showed a partially heterogeneous pattern across tertiles. Patients in the low remnant-C tertile exhibited lower haemoglobin levels compared with both the middle and higher remnant-C groups. Similarly, albumin concentrations were lower in the lower remnant-C group compared with the other tertiles. Consistent with these findings, GNRI values progressively increased across remnant-C tertiles, indicating a more favourable nutritional profile in patients with higher remnant-C levels (90 ± 12 vs. 93 ± 12 vs. 95 ± 10; *P* < .001).

In contrast, nPCR values were comparable across remnant-C tertiles.

Inflammatory burden, as assessed by hs-CRP, did not significantly differ among groups.

### Clinical outcomes and association between remnant cholesterol and cardiovascular risk

During a median follow-up of 31 months [14–60], a total of 328 patients experienced the primary 3-point MACE outcome, corresponding to an event rate of 196.9 per 1000 person-years.

When stratified according to remnant-C tertiles, the proportion of patients experiencing the primary 3-point MACE outcome was 51.0% in the lower tertile, 48.6% in the middle and 58.2% in the higher tertile.

Given the high burden of cardiovascular events observed during follow-up, we subsequently evaluated the association between remnant-C levels and cardiovascular risk through hierarchical Cox proportional hazards models (Table 2).

**Table 2:** Hierarchical Cox proportional hazards regression analysis for 3-point MACE in the study population.

Variables	Remnant-C HR (95% CI)	*P*-value
Unadjusted	1.03 (0.98–1.08)	.110
Model 1: adj. for age and sex	1.07 (1.02–1.12)	.008
Model 2: adj. for age, sex, BMI, diabetes, hypertension, smoking status, history of previous CVD	1.07 (1.02–1.13)	.011
Model 3: Model 2 + LDL	1.06 (1.01–1.12)	.023
Model 4: Model 3 + statin therapy	1.06 (1.01–1.11)	.025

In unadjusted Cox proportional hazards analysis, remnant-C was not significantly associated with the risk of 3-point MACE (HR 1.03, 95% CI 0.98–1.08). However, after adjustment for age and sex, remnant-C emerged as an independent predictor of cardiovascular events (HR 1.07, 95% CI 1.01–1.12). The association remained significant after further adjustment for BMI, diabetes, hypertension, smoking status and previous CVD (HR 1.07, 95% CI 1.02–1.13; *P* = .011). Importantly, remnant-C retained its independent prognostic value even after additional adjustment for LDL-C (HR 1.06, 95% CI 1.01–1.12) and statin therapy (HR 1.06, 95% CI 1.01–1.11) (Table 2).

To further account for the potential impact of nutritional and catabolic status, a sensitivity analysis additionally including both GNRI and nPCR was performed. Notably, remnant-C remained independently associated with 3-point MACE after adjustment for both GNRI and nPCR (HR 1.06, 95% CI 1.00–1.12; *P* = .049).

Restricted cubic spline analysis showed a progressive increase in cardiovascular risk with increasing remnant-C levels, with hazard ratios exceeding 1 at ∼30 mg/dl. No clear threshold effect was observed, supporting an approximately linear association between remnant-C and 3-point MACE risk (Fig. 1).

Multivariable-adjusted survival curves by remnant-C tertiles showed progressive separation of cardiovascular risk over time, with the highest tertile exhibiting the lowest adjusted MACE-free survival although differences did not reach statistical significance (*P* = .169) (Supplementary Fig. S2).

Forest plot analysis of the fully adjusted cardiovascular model confirmed the independent association between remnant-C and 3-point MACE. Diabetes mellitus and previous CVD emerged as the strongest predictors of cardiovascular events whereas LDL-C did not retain independent prognostic significance after multivariable adjustment. Although GNRI differed across remnant-C tertiles, it was not independently associated with the risk of 3-point MACE in the fully adjusted model. Similarly, BMI, smoking, hypertension and sex were not significantly associated with the primary outcome (Fig. 2).

**Figure 2:**
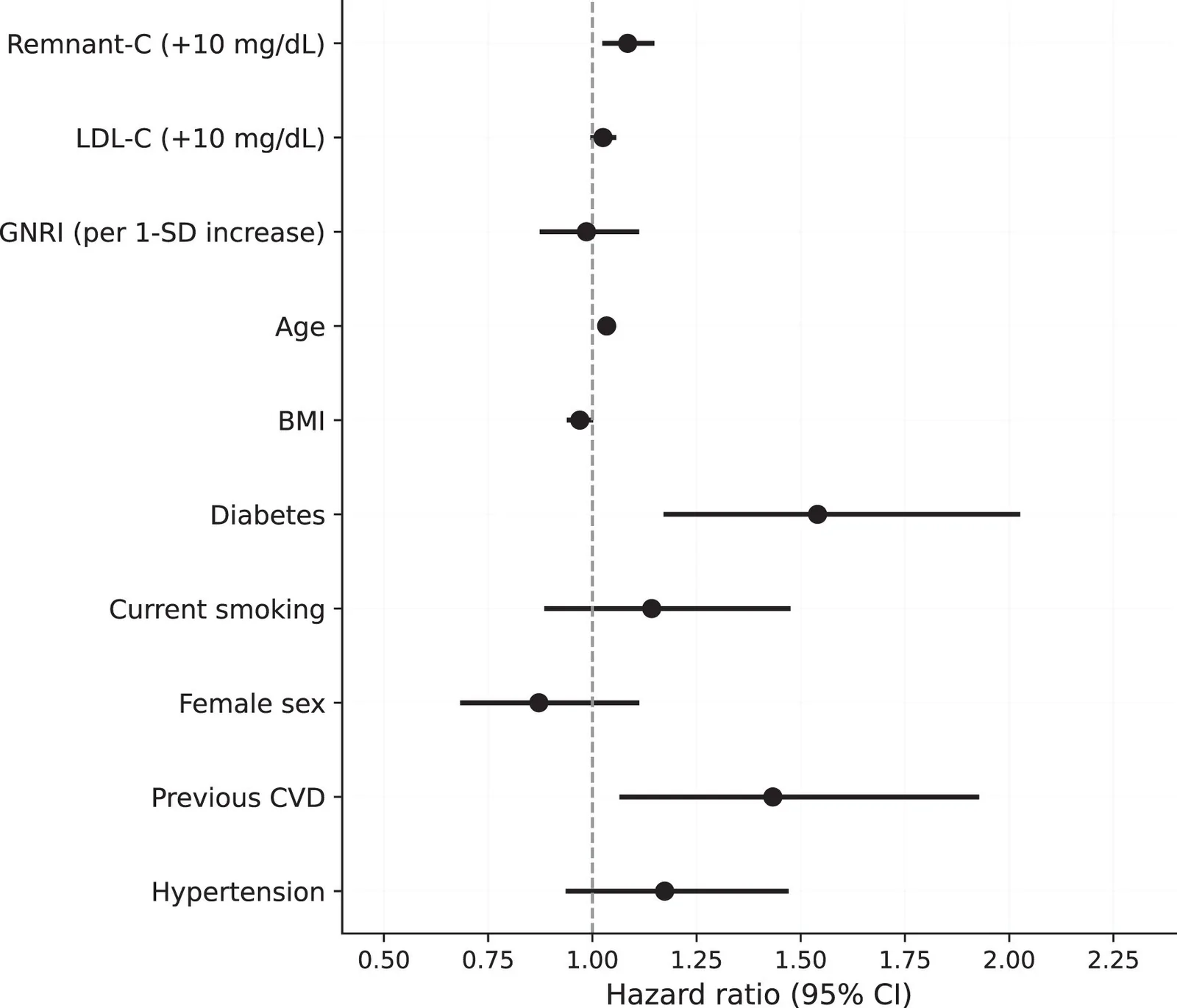
Forest plot of multivariable predictors of 3-point major adverse cardiovascular events (MACE). Forest plot showing hazard ratios (HRs) and 95% confidence intervals derived from the fully adjusted Cox proportional hazards model for 3-point MACE. Remnant cholesterol was analysed per 10 mg/dl increase, whereas the Geriatric Nutritional Risk Index (GNRI) was analysed per 1-standard deviation (SD) increase. The model included age, sex, BMI, diabetes mellitus, smoking status, hypertension, previous cardiovascular disease, LDL-C, and GNRI.

### Association between remnant cholesterol and all-cause mortality

Three hundred seventy-five all-cause deaths occurred during follow-up, corresponding to an event rate of 194.8 per 1000 person-years. The prevalence of all-cause mortality across remnant-C tertiles was 67.3%, 54.8%, and 58.2% in the lower, middle and higher tertile respectively.

In analyses focused on all-cause mortality, remnant-C showed a different pattern of association in respect to cardiovascular outcomes. In unadjusted analysis, higher remnant-C levels were associated with lower mortality risk (HR 0.95, 95% CI 0.89–0.99; *P* = .036). However, this association progressively attenuated after multivariable adjustment and did not remain statistically significant in fully adjusted models (Table 3).

**Table 3:** Hierarchical Cox proportional hazards regression analysis for all-cause mortality in the study population.

Variables	Remnant-C HR (95% CI)	*P*-value
Unadjusted	0.95 (0.89–0.99)	.036
Model 1: adj. for age and sex	0.97 (0.92–1.02)	.156
Model 2: adj. for age, sex, BMI, diabetes, hypertension, smoking status, history of previous CVD	0.95 (0.90–1.01)	.091
Model 3: Model 2 + LDL	0.96 (0.91–1.01)	.105
Model 4: Model 3 + statin therapy	0.96 (0.90–1.01)	.107

Forest plot analysis of the fully adjusted mortality model highlighted a predominant contribution of nutritional and comorbidity burden to mortality risk (Fig. 3). GNRI showed a significant inverse association with all-cause mortality.

**Figure 3:**
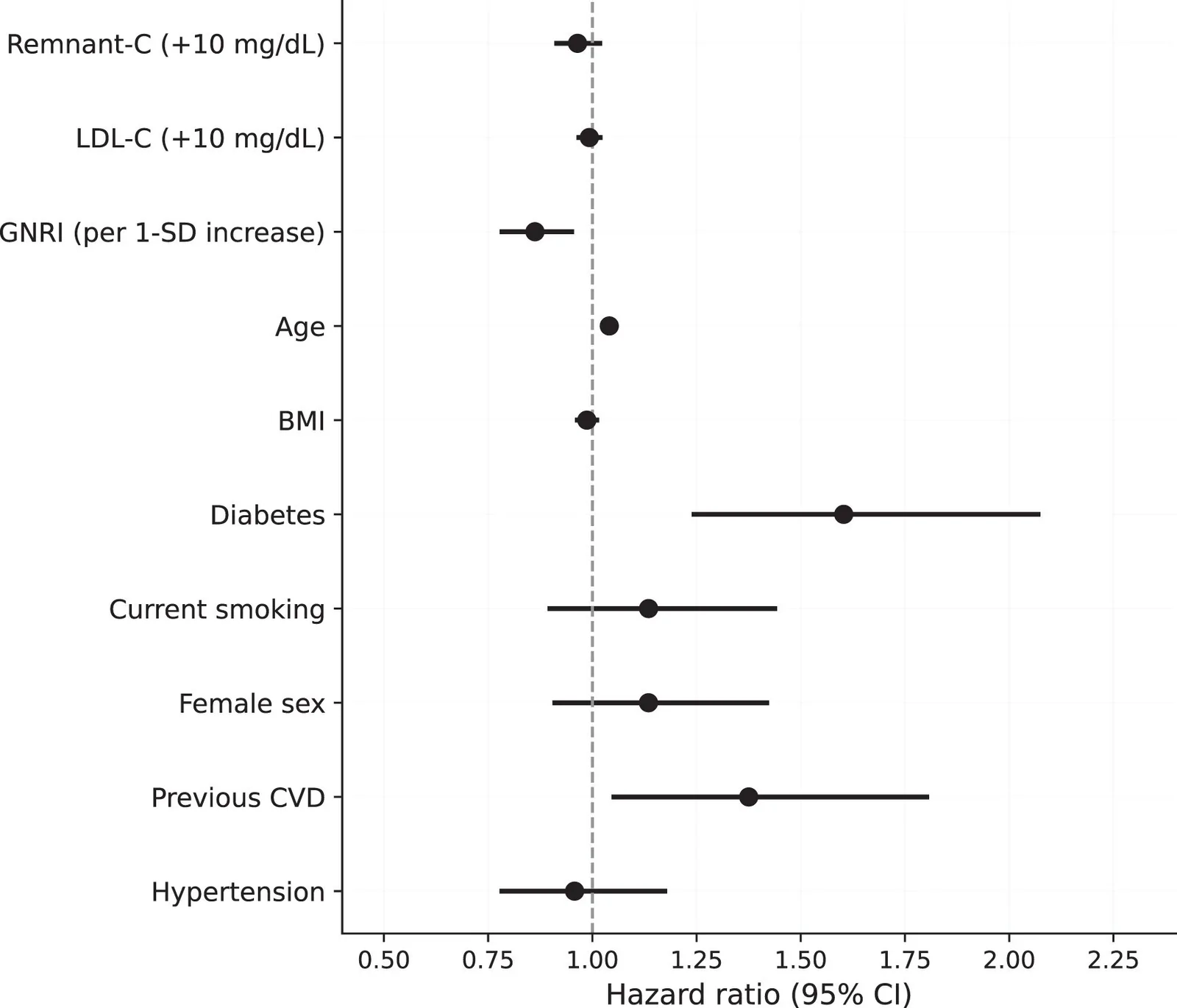
Forest plot of multivariable predictors of all-cause mortality. Forest plot showing hazard ratios (HRs) and 95% confidence intervals derived from the fully adjusted Cox proportional hazards model for all-cause mortality. Remnant cholesterol was analysed per 10 mg/dl increase whereas the GNRI was analysed per 1-standard deviation (SD) increase. The model included age, sex, BMI, diabetes mellitus, smoking status, hypertension, previous cardiovascular disease, LDL-C, and GNRI.

Increasing age and the presence of diabetes were independently associated with higher mortality risk; in contrast, remnant-C and LDL-C did not retain independent prognostic significance after multivariable adjustment.

In a sensitivity analysis, additional adjustment for nPCR yielded materially unchanged results, with both GNRI (per 1-SD increase: HR 0.86, 95% CI 0.76–0.99) and nPCR (HR 0.47, 95% CI 0.29–0.76) remaining independently associated with all-cause mortality.

## DISCUSSION

In this cohort of prevalent dialysis patients, we found an independent association between remnant-C and 3-point MACE, even after adjustment for LDL-C and traditional cardiovascular risk factors, whereas LDL-C itself did not retain an independent prognostic significance. In contrast, remnant-C was not independently associated with all-cause mortality while GNRI emerged as an inverse predictor of mortality risk. Taken together, these findings suggest that, in ESKD, remnant-C may reflect partially distinct biological pathways underlying cardiovascular events and overall survival. Importantly, the simultaneous evaluation of remnant-C, LDL-C and GNRI within the same dialysis cohort allowed us to explore the potentially distinct contribution of residual atherogenic risk and nutritional-frailty pathways to cardiovascular and mortality outcomes.

The association between remnant-C and cardiovascular risk observed in our study is consistent with other data supporting the role of triglyceride-rich lipoproteins and their remnants as contributors to residual atherogenic risk beyond LDL-C [20]. In the general population, elevated remnant-C has been associated with ischaemic heart disease, myocardial infarction and ischaemic stroke independently of conventional lipid parameters [21, 22]. Analyses from the PREDIMED cohort including 6901 individuals at high cardiovascular risk demonstrated that remnant-C independently predicted major cardiovascular events over a median follow-up of 4.8 years (HR 1.21 per 10 mg/dl increase), whereas LDL-C itself did not retain prognostic significance in fully adjusted models [23]. Notably, remnant-C concentrations ≥30 mg/dl identified individuals at increased cardiovascular risk even among subjects with LDL-C within guideline-recommended targets.

More recently, Elías-López *et al*. examined 10 427 individuals with impaired renal function from the Copenhagen General Population Study and reported that higher remnant-C was associated with ASCVD, whereas LDL-C showed no comparable association. This large study provided important evidence that triglyceride-rich lipoprotein cholesterol may better capture residual atherosclerotic risk than LDL-C in individuals with reduced kidney function. However, patients receiving dialysis were excluded, LDL-C was predominantly estimated using the Friedewald equation and nutritional status was not specifically examined [14].

Our findings extend these observations to the dialysis setting, a population characterized by marked alterations in lipid metabolism and exceptionally high cardiovascular risk. Although smaller than that reported in PREDIMED, the effect estimate should be interpreted in relation to the exposure unit. Assuming an approximately log-linear association, the HR of 1.06 per 10 mg/dl corresponds to an estimated HR of 1.25 for a 1 mmol/l (38.7 mg/dl) higher remnant-C concentration. For context, the Cholesterol Treatment Trialists’ Collaboration reported a 22% reduction in major vascular events per 1 mmol/l reduction in LDL-C. Although these estimates are not directly comparable, this comparison suggests that the observed association should not be considered clinically negligible.

The biological plausibility of these findings is supported by the distinct properties of remnant lipoproteins. Although both LDL and remnant particles are apoB-containing and atherogenic, remnant lipoproteins are more cholesterol-rich and can directly penetrate the arterial wall, promoting foam-cell formation and plaque progression [24, 25]. Moreover, dialysis patients exhibit a characteristic dyslipidaemic profile marked by hypertriglyceridaemia, reduced HDL-C, and impaired clearance of triglyceride-rich lipoproteins, favouring remnant accumulation [26]. In this context, remnant-C may better capture the atherogenic burden related to triglyceride-rich lipoproteins than LDL-C alone.

Beyond their direct atherogenic effects, remnant lipoproteins may contribute to residual cardiovascular risk through inflammatory and immune-metabolic pathways. Experimental and translational studies have linked elevated remnant-C to vascular inflammation, innate immune activation, and pro-atherogenic monocyte remodelling [27, 28].

In a mechanistic study conducted in patients with familial dys-beta-lipoproteinaemia, elevated remnant-C was associated with increased arterial 18F-FDG uptake at PET imaging, monocytosis and a pro-adhesive monocyte phenotype [29]. Varbo *et al*. likewise reported associations between remnant-C, ischaemic heart disease and low-grade inflammation, whereas LDL-C was associated only with ischaemic heart disease [30].

In ESKD, characterized by chronic oxidative stress, endothelial dysfunction, and innate immune activation, remnant-rich lipoproteins may contribute to residual cardiovascular risk beyond conventional LDL-mediated atherosclerosis [31, 32].

Our results are also coherent with recent data specifically addressing dialysis populations. A nationwide Korean cohort including more than 20 000 dialysis patients demonstrated that elevated remnant-C was independently associated with myocardial infarction, whereas no clear association was observed for ischaemic stroke [15]. Spline analyses likewise showed a progressive increase in myocardial infarction risk with higher remnant-C levels and no clear threshold effect mirroring our findings of increasing cardiovascular risk above ∼30 mg/dl.

Patients with higher remnant-C showed a less favourable metabolic profile, including higher BMI, a greater prevalence of diabetes and more pronounced dyslipidaemia. However, they were also younger and had higher albumin and GNRI values. This mixed metabolic and nutritional phenotype may partly explain why the association with 3-point MACE became evident only after adjustment for age, sex, and other relevant covariates.

An important aspect of our findings is the lack of independent association between LDL-C and both cardiovascular and mortality outcomes. This observation may appear counterintuitive from the perspective of the general population but it is biologically plausible in ESKD. In dialysis patients, traditional lipid biomarkers are strongly influenced by malnutrition, protein-energy wasting and competing risks, phenomena collectively contributing to the well-described framework of reverse epidemiology [32, 33].

Furthermore, large randomized trials conducted in haemodialysis populations, including 4D and AURORA, failed to demonstrate the same magnitude of cardiovascular benefit from statin therapy observed in non-CKD populations, suggesting that LDL-centred paradigms may incompletely capture cardiovascular risk in ESKD [6, 34]. Thus, the absence of a significant association for LDL-C in our cohort may reflect both the altered lipid biology of advanced kidney disease and the dominant contribution of nontraditional cardiovascular mechanisms.

The divergent findings for MACE and all-cause mortality represent another key aspect of the present study. While remnant-C emerged as an independent determinant of cardiovascular events, nutritional markers appeared more relevant for overall survival. In particular, GNRI and nPCR were inversely associated with all-cause mortality, while BMI showed a pattern consistent with reverse epidemiology. This is biologically plausible, as mortality in dialysis patients reflects not only atherosclerotic burden but also frailty and nutritional status [35–37].

This interpretation is consistent with previous evidence showing that GNRI strongly predicts all-cause mortality in maintenance haemodialysis patients. In a Japanese cohort, GNRI independently predicted all-cause but not cardiovascular mortality. Likewise, in the original RISCAVID cohort, low GNRI strongly predicted overall mortality, whereas cardiovascular events did not differ across GNRI categories. Together, these findings support the concept that GNRI primarily reflects the nutrition-frailty axis rather than atherosclerotic cardiovascular risk [38, 39].

These findings suggest that cardiovascular events and all-cause mortality may be driven by partly distinct pathways in ESKD. Remnant-C appears more closely linked to residual atherogenic and vascular inflammatory risk, whereas GNRI may primarily reflect the nutrition-frailty axis. This may explain why remnant-C independently predicted cardiovascular events, while GNRI emerged as a stronger determinant of survival.

The present findings may have implications for cardiovascular risk stratification, with remnant-C serving as a complementary marker of residual atherogenic risk beyond LDL-C rather than an established therapeutic target. No intervention has yet demonstrated that specifically lowering remnant-C improves cardiovascular outcomes in haemodialysis. Management should therefore focus on correction of secondary causes of hypertriglyceridaemia and individualized dietary and lifestyle measures, balanced against the risk of protein-energy wasting. Pharmacological options remain limited: routine initiation of statin therapy is not recommended in prevalent dialysis patients, while fibrates have limited applicability in ESKD because of safety concerns [40, 41]. In the PISCES trial, n-3 polyunsaturated fatty acid supplementation reduced serious cardiovascular events although whether this benefit was mediated by reductions in triglyceride-rich lipoproteins or remnant-C remains unknown [42]. Dedicated trials are therefore needed before remnant-C can be considered a therapeutic target.

Several limitations should be acknowledged. First, remnant-C was calculated rather than directly measured and standardized assays for remnant lipoproteins are still not routinely available in clinical practice. Second, we lacked information regarding apoB and longitudinal changes in lipid parameters during follow-up. Third, the study population consisted of prevalent dialysis patients, potentially introducing survivor bias and limiting generalizability to incident ESKD populations. Despite these limitations, our study also has several strengths. We evaluated a wide cohort of patients receiving maintenance dialysis with detailed clinical and nutritional phenotyping. In addition, the relatively long follow-up and the combined evaluation of both cardiovascular and mortality outcomes provided the opportunity to explore the potentially divergent biological pathways underlying residual cardiovascular risk and overall survival in ESKD.

In conclusion, remnant-C was independently associated with cardiovascular events but not all-cause mortality, whereas GNRI was inversely associated with mortality risk. These findings support distinct contributions of residual atherogenic risk and nutrition-frailty pathways to cardiovascular and survival outcomes in ESKD and highlight remnant-C as a complementary cardiovascular risk marker beyond LDL-C.

## Supplementary Material

sfag337_Supplemental_Files

## Data Availability

Data that support the findings of this study are available from the corresponding author upon reasonable request.
