# Supplementary material for: Remnant cholesterol and cardiovascular risk in maintenance haemodialysis
Source: Clin Kidney J. 2026 Sep 25;19(10):sfag337. doi: 10.1093/ckj/sfag337 (PMC13655738; doi:10.1093/ckj/sfag337)
Supplement: Supplementary file 1 [file sfag337_supplemental_files.zip › Figure S1.pdf]

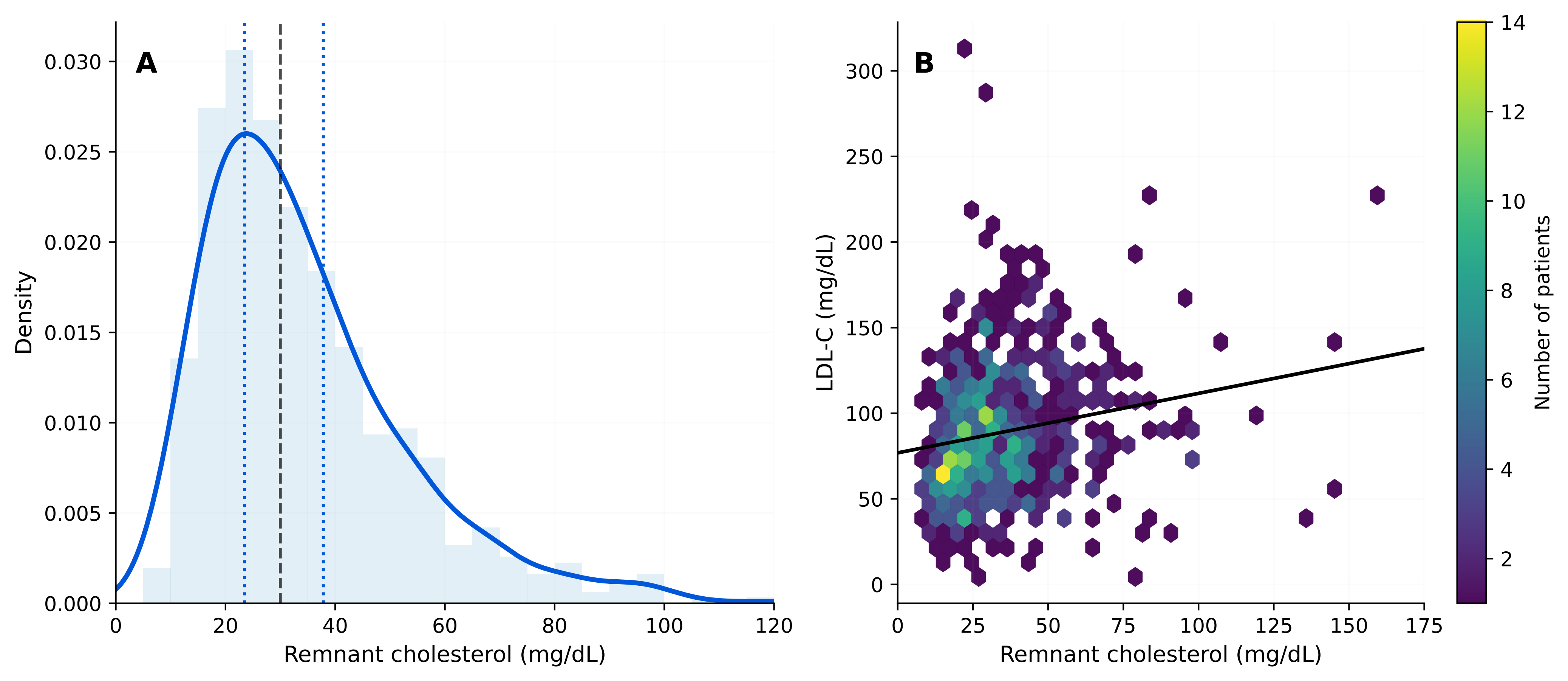
A
Density
Remnant cholesterol (mg/dL)
B
LDL-C (mg/dL)
Remnant cholesterol (mg/dL)
Number of patients
