# Supplementary material for: Remnant cholesterol and cardiovascular risk in maintenance haemodialysis
Source: Clin Kidney J. 2026 Sep 25;19(10):sfag337. doi: 10.1093/ckj/sfag337 (PMC13655738; doi:10.1093/ckj/sfag337)
Supplement: Supplementary file 1 [file sfag337_supplemental_files.zip › Figure S2.pdf]

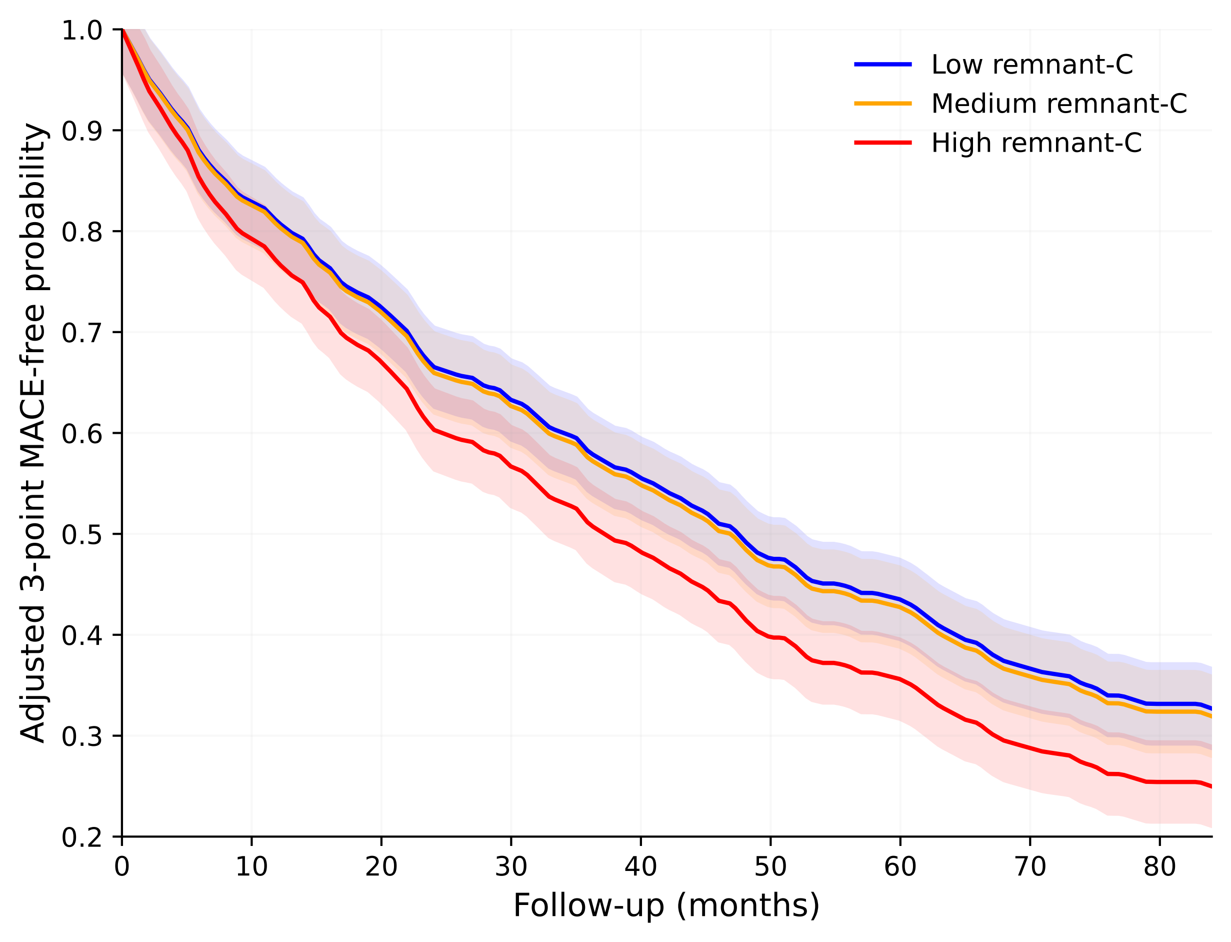

Low remnant-C
Medium remnant-C
High remnant-C
Adjusted 3-point MACE-free probability
1.0
0.9
0.8
0.7
0.6
0.5
0.4
0.3
0.2
0
10
20
30
40
50
60
70
80
Follow-up (months)
